# The associations between maternal and fetal exposure to endocrine-disrupting chemicals and asymmetric fetal growth restriction: a prospective cohort study

**DOI:** 10.3389/fpubh.2024.1351786

**Published:** 2024-04-11

**Authors:** Subeen Hong, Byung Soo Kang, Oyoung Kim, Sangeun Won, Hyeon Soo Kim, Jeong Ha Wie, Jae Eun Shin, Sae Kyung Choi, Yun Sung Jo, Yeon Hee Kim, Mihi Yang, Huiwon Kang, Dong-Wook Lee, In Yang Park, Joong Shin Park, Hyun Sun Ko

**Affiliations:** ^1^Department of Obstetrics and Gynecology, Seoul St. Mary’s Hospital, College of Medicine, The Catholic University of Korea, Seoul, Republic of Korea; ^2^Department of Obstetrics and Gynecology, Eunpyeong St. Mary's Hospital, College of Medicine, The Catholic University of Korea, Seoul, Republic of Korea; ^3^Department of Obstetrics and Gynecology, Bucheon St. Mary’s Hospital, College of Medicine, The Catholic University of Korea, Seoul, Republic of Korea; ^4^Department of Obstetrics and Gynecology, Incheon St. Mary’s Hospital, College of Medicine, The Catholic University of Korea, Seoul, Republic of Korea; ^5^Department of Obstetrics and Gynecology, St. Vincent’s Hospital, College of Medicine, The Catholic University of Korea, Seoul, Republic of Korea; ^6^Department of Obstetrics and Gynecology, Uijeongbu St. Mary’s Hospital, College of Medicine, The Catholic University of Korea, Seoul, Republic of Korea; ^7^College of Pharmacy, Sookmyung Women’s University, Seoul, Republic of Korea; ^8^Goodbeing Center Co. Ltd., Seoul, Republic of Korea; ^9^Department of Occupational & Environmental Medicine, Inha University Hospital, Inha University, Incheon, Republic of Korea; ^10^Department of Obstetrics and Gynecology, Seoul National University College of Medicine, Seoul, Republic of Korea

**Keywords:** endocrine disruptors, bisphenol-A, monoethyl phthalates, perfluorooctanoic acid, fetal growth restriction, placental insufficiency

## Abstract

Recent evidence has revealed associations between endocrine-disrupting chemicals (EDCs) and placental insufficiency due to altered placental growth, syncytialization, and trophoblast invasion. However, no epidemiologic study has reported associations between exposure to EDCs and asymmetric fetal growth restriction (FGR) caused by placenta insufficiency. The aim of this study was to evaluate the association between EDC exposure and asymmetric FGR. This was a prospective cohort study including women admitted for delivery to the Maternal Fetal Center at Seoul St. Mary’s Hospital between October 2021 and October 2022. Maternal urine and cord blood samples were collected, and the levels of bisphenol-A (BPA), monoethyl phthalates, and perfluorooctanoic acid in each specimen were analyzed. We investigated linear and non-linear associations between the levels of EDCs and fetal growth parameters, including the head circumference (HC)/abdominal circumference (AC) ratio as an asymmetric parameter. The levels of EDCs were compared between fetuses with and without asymmetric FGR. Of the EDCs, only the fetal levels of BPA showed a linear association with the HC/AC ratio after adjusting for confounding variables (*β* = 0.003, *p* < 0.05). When comparing the normal growth and asymmetric FGR groups, the asymmetric FGR group showed significantly higher maternal and fetal BPA levels compared to the normal growth group (maternal urine BPA, 3.99 μg/g creatinine vs. 1.71 μg/g creatinine [*p* < 0.05]; cord blood BPA, 1.96 μg/L vs. −0.86 μg/L [*p* < 0.05]). In conclusion, fetal exposure levels of BPA show linear associations with asymmetric fetal growth patterns. High maternal and fetal exposure to BPA might be associated with asymmetric FGR.

## Introduction

Exposure to endocrine-disrupting chemicals (EDCs) has raised major public health concerns, especially during the coronavirus disease 2019 (COVID-19) pandemic (1–5). EDCs can affect the action of hormones and exhibit a non-linear dose–response relationship in the human body related to various diseases, such as reproductive disorders, neurodevelopmental disorders, metabolic disorders, and certain types of cancer (6–10). In particular, maternal exposure to EDCs during pregnancy is related not only to maternal disorders but also abnormal fetal development (11–14).

Bisphenols, phthalates, and persistent organic pollutants are EDCs that humans are frequently exposed to and that have been extensively studied for their effects on fetal growth (15–24). Some meta-analysis or systematic review studies have reported negative correlations between bisphenols (15), phthalates (19), per-and polyfluoroalkyl substances (22, 24), and birth weight, while others have failed to find a correlation (17) or have reported a reverse correlation (16).

FGR is a common manifestation and clinically important in obstetrics as a cause of perinatal morbidities and mortalities (25–27). Although the etiologies of FGR vary, placenta insufficiency is a major one. Placenta-mediated FGR is characterized by an asymmetric pattern of fetal growth, and it shares a pathological process with hypertensive disorders of pregnancy (28, 29). Asymmetric growth patterns are thought to occur when chronic hypoxia and malnutrition due to placental insufficiency lead to adaptation to chronic hypoxia. This adaptation involves a brain-sparing effect, where blood flow to the brain increases, resulting in an increase in head circumference (HC), while the abdominal circumference (AC) of the fetus becomes relatively small (30–32).

There are various mechanisms proposed for how EDCs may affect fetal growth. Including alterations in hormone homeostasis, inflammation with oxidative stress, and epigenetic changes (33). EDCs may affect not only the fetus directly but also the size and function of the placenta (34, 35). Early exposure to these chemicals has been linked to altered syncytialization, trophoblast invasion, and spiral artery remodeling, which can result in placental insufficiency and subsequently lead to FGR through chronic hypoxia and malnutrition (36–39). Based on the laboratory evidence, EDCs could induce FGR through placental insufficiency, and it could result in fetal asymmetric growth.

Despite this evidence, most epidemiological studies to date have focused on the associations between different EDCs and birth weight itself. In addition, information on fetal exposures is limited, and population-based studies on the association between fetal asymmetric growth and EDC exposures are lacking. We aimed to investigate the potential effects of maternal and fetal exposure to EDCs on the asymmetric pattern of FGR.

## Methods

### Study design and population

This analysis considered data from a prospective cohort study designed to examine fetal–maternal exposure to EDCs during the COVID-19 pandemic and the association between EDC exposure and obstetric complications. The cohort study enrolled 323 women admitted for delivery to the Maternal Fetal Center at Seoul St. Mary’s Hospital between October 2021 and October 2022. We collected anthropometric measurements, socioeconomic status, medical history, and maternal urine samples after obtaining informed consent and cord blood samples after delivery. Ultimately, 146 mother–child pairs with singleton pregnancies with adequate amounts of maternal urine and cord blood samples for analyzing EDCs and adequate data of fetal growth at admission were included in the study. We evaluated the distributions; fetal–maternal transfer of EDCs; and the association between levels of EDCs in each specimen and fetal growth parameters, including asymmetric indices. In addition, the study population was divided into groups according to the presence of fetal growth restriction or asymmetricity, and the levels of EDCs were compared between the groups. This study was conducted after informed consent was obtained from all participants and was approved by the institutional review board (IRB) of the Catholic Medical Center in South Korea (IRB no. XC21ONDI0125).

### Assessment of EDC exposure

Maternal urine samples were obtained after hospitalization for delivery, and cord blood was collected directly from the cord immediately after birth. Cord blood serum was fractioned by centrifugation at 3000 rpm for 10 min. The samples were stored at −80°C until analysis. We selected a set of environmentally disruptive chemicals for assessment, focusing on bisphenol-A (BPA), monoethyl phthalates (MEPs), and perfluorooctanoic acid (PFOA); it is believed that pregnant women are commonly exposed in daily lives to these chemicals, which have been extensively studied during the COVID-19 pandemic period (3, 4) but there are controversial results in the relation between these EDC exposure and fetal growth (15–24). The levels of EDCs were analyzed with ultra–performance liquid chromatography–tandem mass spectrometry (UPLC-MS/MS). In detail, BPA and MEP were analyzed with Agilent 1,260 Infinity UPLC system (Agilent Technologies, Santa Clara, CA) and MS, the Agilent Triple Quadrupole 6,460 system with a specialized type of ESI interface. PFOA was analyzed using a different system, i.e., UPLC of Thermofisher Scientific Ultimate (Thermo Fisher Scientific, Waltham, MA). and Bruker EVOQ Qube LC-Triple Quadrupole (Bruker corporation, Billerica, MA), equipped to avoid fluoride contamination. The limit of detection (LOD) for BPA, MEP, and PFOA were 0.1, 0.1, and 0.01 μg/L, respectively. For statistical analyses, undetected levels of EDCs were replaced with a value of one-half of the minimum detected level of EDCs (40). To adjust the urine dilutions, metabolite levels were divided by creatinine (Cr) levels, then analyzed using an automatic biomedical analyzer (HITACHI 7020; Hitachi, Ltd., Tokyo, Japan). Detailed methods and calibration curves for analyzing the EDCs are described in the Supplementary materials.

### Definition of fetal growth restriction

The fetal growth parameters measured using transabdominal ultrasound by skilled physicians after hospitalization for delivery. The median gestational age at the time of ultrasound was 38.3 weeks of gestation. Fetal growth parameters include biparietal diameter (BPD), head circumference (HC), abdominal circumference (AC), femur length (FL), and estimated fetal weight (g). Since these parameters vary greatly depending on the gestational age, *z*-scores were calculated using the Intergrowth-21 chart (41, 42). Asymmetric growth parameters were assessed using the HC/AC ratio (43, 44), which was calculated as the ratio of HC (mm) to AC (mm). Fetal growth restriction was defined according to the Society for Maternal–Fetal Medicine guidelines as an AC measurement below the 10th percentile or an estimated fetal weight below the 10th percentile (45). Asymmetric growth was defined as an HC/AC ratio above the 95th percentile based on the criteria published by Campbell and Thoms (43).

#### Covariates

We collected maternal demographic characteristics and socioeconomic status indicators, including maternal age, parity, pre-pregnancy body mass index (BMI), pre-pregnancy smoking, pre-pregnancy alcohol consumption, pre-existing hypertension, pre-existing diabetes mellitus, gestational diabetes mellitus, and pregnancy-associated hypertension. Obstetric and delivery outcomes were acquired, including gestational age at delivery, cesarean section delivery, fetal sex, birth weight, and neonatal intensive care unit admission. We selected five confounding variables according to a rule-of-thumb of multivariable linear regression analysis (46). The adjusted confounders that might influence fetal growth parameters included maternal age, pre-pregnancy BMI, pre-pregnancy smoking, gestational age at ultrasound examination, and fetal sex.

### Statistical analysis

Continuous data are presented as mean ± standard deviation values and categorical variables are presented as a number (%). The analyses from urine samples were conducted using Cr-adjusted levels of EDCs. To assess the distribution of environmental hormones, the geometric means (GMs) of BPA, MEP, and PFOA levels were calculated. For further statistical analyses, the Cr-adjusted levels of EDCs were transformed by Log2-transformation because they were not normally distributed. For the analysis of maternal to fetal exposure of EDCs, the correlation between the level of EDCs in maternal urine and those in fetal cord blood was assessed using a generalized additive model and linear regression model, and Pearson’s correlation coefficient was calculated.

To investigate the association between levels of EDCs and fetal growth parameters, we first used generalized additive models to assess the linearity or non-linearity of the associations. We then used multivariable linear regression analysis or generalized additive model analysis to identify any significant relationships between EDC levels and fetal growth parameters, while adjusting for confounding variables. The regression coefficients from these analyses represented the difference in growth parameters per two-fold increase in EDC levels. To examine the association between EDC levels and fetal growth restriction (FGR), we divided the study population into FGR and non-FGR groups, then compared the concentrations of EDCs using the Mann–Whitney *U* test. The same methods were used to compare EDC levels between the FGR with asymmetry group and the non-FGR group. We conducted these analyses using IBM SPSS Statistics version 25.0 software (IBM Corp., Armonk, NY, USA) and R version 4.2.1^1^ (R Foundation for Statistical Computing, Vienna, Austria).

## Results

### Characteristics of the total study population

A total of 146 women were included in this study, and their baseline characteristics, obstetric history, and delivery outcomes are presented in Table 1. The median maternal age was 35 years, and the median BMI was 21.2 kg/m^2^. The median gestational age at delivery was 38.6 weeks, and most of the study population delivered after 37 weeks of gestation. The median birth weight was 3,118 g, and 92.5% of neonates had an adequate birth weight, ranging from 2,500 g to <4,000 g.

**Table 1:** Baseline characteristics of total study population.

	*N* (%)	Median (IQR)
Total	146 (100.0)	
Maternal age (years)		35 (32, 38)
Age < 35	73 (50.0)	
Age ≥ 35	73 (50.0)	
Parity		
Nulliparity	86 (58.9)	
Primiparity or multiparity	60 (41.1)	
Prepregnancy BMI (kg/m^2^)		21.2 (19.4, 23.6)
Normal (BMI <23)	104 (71.2)	
Overweight (25 > BMI ≥ 23)	21 (14.4)	
Obese (BMI ≥ 25)	21 (14.4)	
Pre-pregnancy smoking		
No	135 (92.5)	
Yes	11 (7.5)	
Pre-pregnancy alcohol consumption		
No	49 (33.6)	
Yes	97 (66.4)	
Preexisting hypertension		
No	140 (95.9)	
Yes	6 (4.1)	
Gestational diabetes mellitus		
No	106 (72.6)	
Yes	40 (27.4)	
Pregnancy associated hypertension		
No	141 (96.6)	
Yes	5 (3.4)	
GA at delivery (weeks)		38.6 (38.0, 39.6)
Preterm birth (GA < 37 weeks)	9 (6.2)	
Full-term birth (GA ≥ 37 weeks)	137 (93.8)	
Mode of Delivery		
Vaginal	61 (41.8)	
Cesarean	85 (58.2)	
Sex		
Boys	58 (39.7)	
Girls	88 (60.3)	
NICU admission		
No	127 (87.0)	
Yes	19 (13.0)	
Birthweight (g)		3,118 (2,838, 3,350)
<2,500	10 (6.8)	
2,500–3,999	135 (92.5)	
≥4,000	1 (0.7)	

IQR, interquartile range; BMI, body mass index; GA, gestational age; NICU, neonatal intensive care unit.

### The distribution of BPA, MEP, and PFOA levels in maternal urine and cord blood

The distribution of BPA, MEP, and PFOA levels in maternal urine and cord blood is shown in Supplementary Table S1. BPA was detected in 93.2 and 91.1% of maternal urine and cord blood samples, respectively. The geometric mean for urine BPA was 1.220 μg/g creatinine, while that for cord blood was 0.751 μg/L. MEP was detected in 91.1 and 77.4% of maternal urine and cord blood samples, respectively. The geometric mean for urine MEP was 10.523 μg/g creatinine, while that for cord blood was 0.106 μg/L. PFOA was detected in 49.3 and 100% of maternal urine and cord blood samples, respectively. The geometric mean for urine PFOA was 0.026 μg/g creatinine, while that for cord blood was 2.315 μg/L.

### The correlation of BPA, MEP, and PFOA levels in maternal urine and cord blood

**Figure 1:**
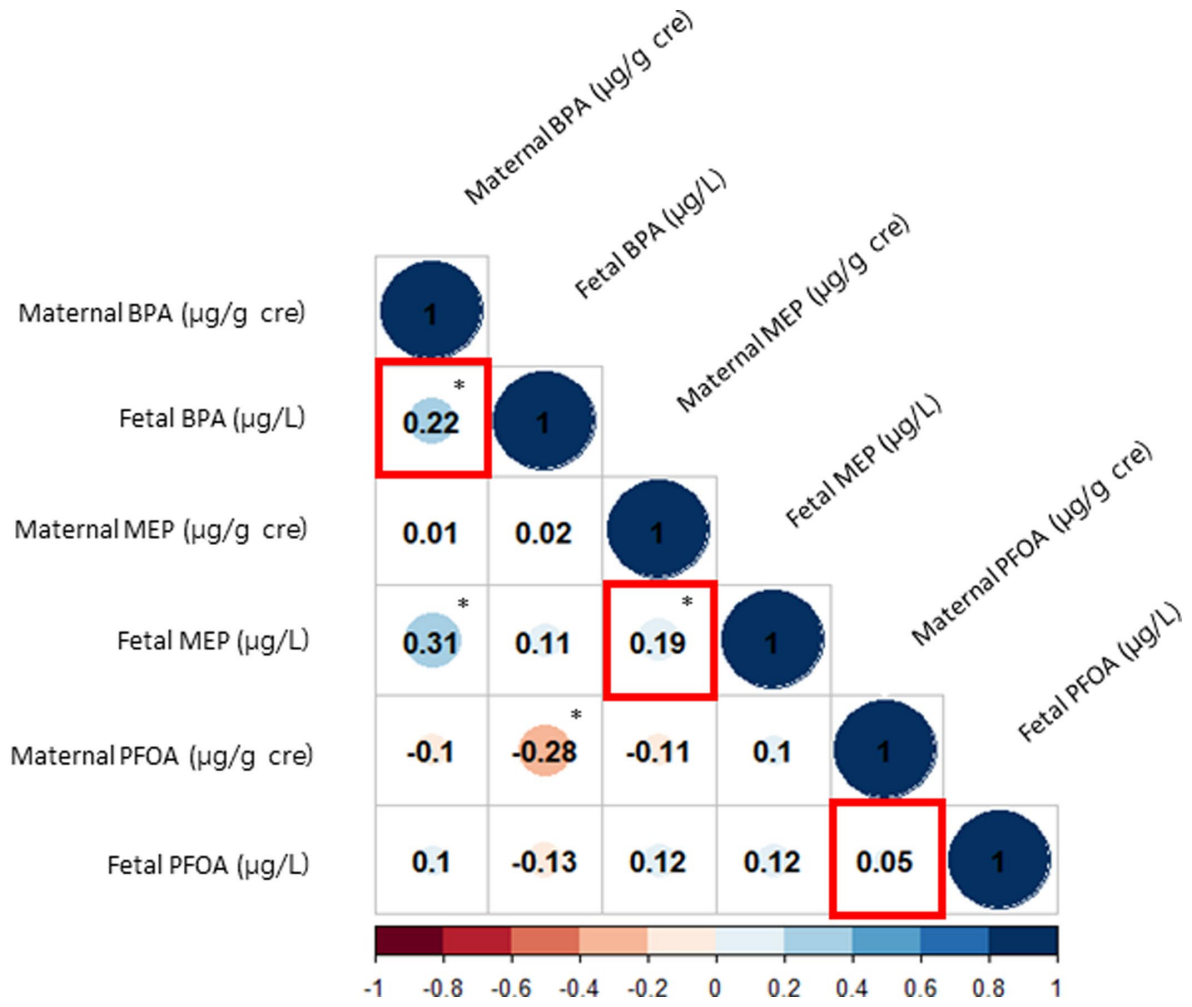
Correlations between EDCs in maternal urine and fetal cord blood. The red squares represent the correlations between maternal urine and fetal cord blood for each EDC. The size of the blue circles indicates the strength of the positive relationship, and the size of the red circles indicates the strength of the negative relationship. The asterisks denote statistically significant associations. EDC, endocrine disrupting chemicals; BPA, bisphenol-A; MEP, monoethyl phthalate; PFOA, perfluorooctanoic acid.

Figure 1 shows the correlation between each sample. The Pearson correlation coefficient between maternal urine BPA and fetal cord blood BPA was 0.22, while that between maternal urine MEP and fetal cord blood MEP was 0.19, indicating a statistically significant positive correlation (both *p* < 0.05). However, there was no significant correlation among PFOA levels between maternal urine and fetal cord blood (correlation coefficient, 0.05; *p* = 0.583). A linear association between maternal urine and cord blood samples in BPA and MEP was confirmed by both non-linear and linear association analyses using a generalized additive model and linear regression model, respectively (Supplementary Figure S1).

### Association between EDC exposure and asymmetric fetal growth restriction

**Figure 2:**
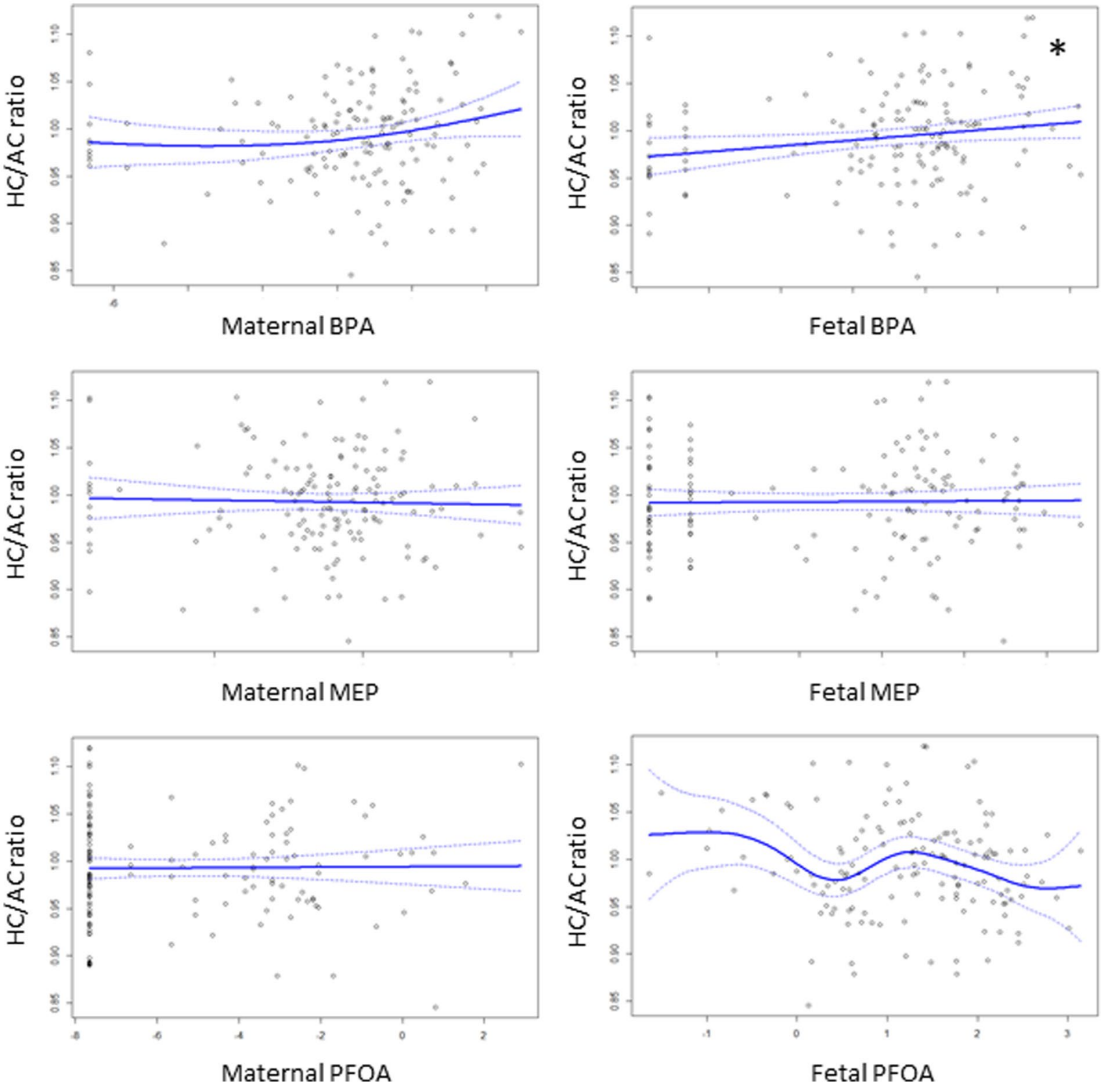
Associations of the levels of EDCs with HC/AC ratio using generalized additive model. The asterisks denote statistically significant associations.

Figure 2 shows associations between the levels of EDCs and the HC/AC ratio as an asymmetric growth parameter. Among the EDCs, fetal cord blood BPA showed a positive linear association with the HC/AC ratio (effective degree of freedom, 1; *R*^2^ = 0.082; *p* < 0.05). After analysis using a multivariable linear regression model, a positive linear association was still observed between cord blood BPA and the HC/AC ratio after adjusting for confounding variables (*β =* 0.003, *p* < 0.05) (Table 2). The results of comparing the EDC levels between groups are presented in Table 3. There were no significant differences in the levels of EDCs between the FGR and non-FGR groups. However, significant differences in BPA levels were observed between the asymmetric FGR and non-FGR groups. The asymmetric FGR group showed significantly higher maternal and fetal BPA levels compared to the control group (maternal urine BPA, 3.99 μg/g of Cr vs. 1.71 μg/g of Cr, *p* < 0.05; cord blood BPA, 1.96 μg/L vs. 0.86 μg/L, *p* < 0.05). Considering baseline characteristics between asymmetric FGR and non-FGR groups, there were no significant differences in other characteristics except for estimated fetal weight and birth weight (Supplementary Table S2).

**Table 2:** Associations of the levels of EDCs with HC/AC ratio using multivariable linear regression model.

	HC/AC ratio		
	*β*	95% CI	*p*-value
Maternal BPA	0.0027	−0.0006, 0.0059	0.106
Fetal BPA	**0.0028**	**0.0001, 0.0055**	**0.043**
Maternal MEP	−0.0002	−0.0028, 0.0025	0.908
Fetal MEP	0.0003	−0.0022, 0.0028	0.783
Maternal PFOA	−0.0002	−0.0033, 0.0028	0.888
Fetal PFOA	−0.0079	−0.0167, 0.0010	0.081

The bold font means remained statistically significance after adjusting for maternal age, body mass index, past smoker, gestational age at exam, and fetal sex. EDC, endocrine disrupting chemicals; HC, head circumference; AC, abdominal circumference; CI, confidence interval; BPA, bisphenol-A; MEP, monoethyl phthalate; PFOA, perfluorooctanoic acid.

**Table 3:** Differences in EDC concentrations between non-FGR group and FGR group, or between non-FGR group and FGR with asymmetry group.

	Non-FGR (*n* = 129)	FGR (*n* = 17)	*p*-value^a^	FGR with asymmetry (*n* = 9)	*p-* value^b^
Maternal BPA	1.711 (0.670, 3.455)	3.380 (1.047, 6.368)	0.118	**3.996 (2.480, 16.018)**	**0.013**
Fetal BPA	0.858 (0.278, 1.589)	0.901 (0.379, 5.615)	0.400	**1.960 (0.793, 6.526)**	**0.036**
Maternal MEP	15.050 (6.015, 36.134)	19.342 (1.966, 53.314)	0.963	19.540 (1.781, 54.250)	0.935
Fetal MEP	0.280 (0.010, 0.701)	0.010 (0.005, 0.559)	0.247	0.010 (0.005, 0.567)	0.302
Maternal PFOA	0.010 (0.005, 0.130)	0.005 (0.005, 0.080)	0.149	0.005 (0.005, 0.005)	0.060
Fetal PFOA	2.440 (1.485, 3.880)	2.540 (1.209, 4.035)	0.791	2.540 (1.209, 3.295)	0.515

^a^Comparison between FGR group and Non-FGR group. ^b^Comparison between FGR with asymmetry group and Non-FGR group. FGR group included fetuses with FGR with asymmetric growth and FGR with symmetric growth. EDC, endocrine disrupting chemicals; FGR, fetal growth restriction; BPA, bisphenol-A; MEP, monoethyl phthalate; PFOA, perfluorooctanoic acid. The bold value means remained statistically significance.

### Association between EDC exposure and fetal growth parameters

When examining the relationship between each growth parameter and EDC levels using generalized additive modeling, we identified significant linear associations between the maternal BPA level and HC and between the maternal MEP level and FL, respectively, while the fetal PFOA level showed a non-linear association with AC (Supplementary Figure S2, all *p* < 0.05) After multivariable linear regression analysis, positive linear associations remained between the maternal MEP level and FL *z*-score (*β =* 0.067, *p* < 0.05) (Supplementary Table S3). Fetal PFOA also showed a significant non-linear association with AC after adjusting for confounding factors (effective degree of freedom, 6.545; *R*^2^ = 0.167; *p* < 0.05). Statistical values for the generalized additive model are presented in Supplementary Table S4.

## Discussion

### Main findings

Our study was conducted to investigate the associations between asymmetric fetal growth restriction and EDC exposure. According to our findings, there was a statistically significant positive correlation between the asymmetric growth indicator HC/AC ratio and the fetal cord blood BPA level. Furthermore, in cases of asymmetric FGR, both maternal urine and cord blood samples showed significantly higher BPA concentrations compared to those of the normal growth group. Based on these results, it can be inferred that elevated BPA levels in fetuses, resulting from maternal BPA exposure, are associated with asymmetric FGR.

### Interpretation

While numerous epidemiologic studies have suggested a negative impact of BPA on fetal growth (47–54), limited research has been conducted on its relationship with asymmetric fetal growth restriction. Most studies have examined associations between BPA and birth weight itself (54), with only a few investigating postnatal asymmetric parameters, such as the ponderal index (17, 55–58). The ponderal index is a postnatal asymmetric growth parameter used to assess the body composition of infants that provides a convenient value derived from each infant’s weight and height; to date, it has been studied in relation to environmental hormones, unlike prenatal indicators such as HC/AC or Doppler indices. Some studies have reported no association between the ponderal index and BPA exposure (17, 55, 56) while others have reported an association between higher levels of BPA and increased ponderal index values (57, 58).

In our study, we used the prenatal HC/AC ratio as an asymmetric growth indicator, reflecting the brain-sparing effect in growth restricted fetuses and reflecting the prenatal asymmetric growth patterns associated with adverse outcomes (31, 32, 59). Although there is currently a lack of research on the relationship between prenatal asymmetric growth indicators and EDCs, some studies have reported associations between BPA exposure during the fetal period and smaller AC and larger HC measurements (16, 53, 60–62). For instance, Zhou et al. reported that higher BPA levels were associated with decreased AC and noted a sex-specific correlation between head circumference and BPA, where increased BPA levels were associated with larger head sizes in girls (16). They explained the observed differences in the effects of BPA on males and females by highlighting the influence of certain hormones and epigenetic mechanisms. A recent study by Uldbjerg et al. also reported a negative association between BPA level and AC (60). However, there are also numerous studies that do not show an association between BPA level and a smaller AC or larger HC, indicating the need to conduct further research in this area (53, 61, 62).

Asymmetric FGR is believed to be attributed to placental insufficiency and the brain-sparing effect in growth restricted fetuses (63, 64). Several *in vitro* studies have shed light on the impact of BPA on placental insufficiency. It has been documented that BPA can induce alterations in the size and morphology of the placenta and exert a negative influence on spiral artery remodeling (36–38). Moreover, BPA has been shown to have detrimental effects on trophoblast invasion, syncytiotrophoblast differentiation, and syncytiotrophoblast zone size, which are critical for proper placental function (39, 65). BPA has also been found to induce oxidative stress, a known contributor to placental insufficiency (66, 67). Additionally, EDCs like BPA can interfere with the expression of crucial nutrition transporters like amino-acid transporters, thereby disrupting the exchange of nutrients (68). Recent research has highlighted an association between BPA and the antiangiogenic factor sFlt-1/PlGF, which plays a role in placental insufficiency (69, 70). Taken together, it is hypothesized that BPA’s potential impact on the placenta may be a contributing factor to the observed asymmetric pattern of fetal growth restriction, as demonstrated in our study.

There exist several epidemiological studies suggesting that MEP and PFOA may contribute to fetal growth impairment (56, 71–76). However, in our study, we did not observe an association between these two EDCs and fetal growth restriction. Nevertheless, MEP showed a positive association with FL, while PFOA exhibited a non-linear association with AC. These findings differ from those of some previous studies where MEP has been reported to be associated with smaller HCs (56, 71, 77). However, multiple studies have also reported no significant relationship between MEP concentrations and fetal growth parameters (53, 60, 61, 70, 78, 79). Similarly, the relationship between PFOA and fetal growth parameters, such as HC and AC, has been investigated in several studies whose results were inconsistent (75, 80, 81). The discrepancies may be due to differences in study methodologies, such as different sampling times or types and diverse population groups.

We assessed the exposure levels of BPA, MEP, and PFOA during the COVID-19 pandemic. Despite the assumption that plastic usage would increase during the COVID-19 pandemic, leading to greater exposure to BPA and phthalates, our results showed similar or even lower levels of BPA, MEP, and PFOA compared to earlier studies conducted in South Korea (58, 82–86). It is speculated that, although plastic usage or exposure to environmental hazards may have increased, many products used by mothers are now labeled as BPA-free, and there have been environmental regulations on BPA, phthalates, and PFOA, which may have contributed to the decreased levels of exposure. In addition, we observed a correlation between BPA and MEP levels in both maternal samples and cord blood samples. This finding is consistent with existing knowledge that many environmental chemicals can pass through the placenta and contribute to exposure *in utero* (87–90).

### Strengths and limitations

Our study holds significance as the first investigation to explore the association between asymmetric fetal growth restriction and EDC exposure. It was conducted prospectively, enabling the examination of EDC correlations between maternal urine and cord blood samples and their relationship with fetal growth. Additionally, the present study was conducted during the COVID-19 pandemic, providing information about environmental hormone exposure during this period.

However, our study also has several limitations. Recently, Doppler parameters have been recognized as better predictors of FGR outcomes than the HC/AC ratio, yet our study did not include severe FGR cases exhibiting significant Doppler abnormalities. In addition, we did not provide additional information related to placental dysfunction, such as placental biopsy results or sFlt-1/PlGF results. The number of FGR cases was limited, which prevented us from presenting adjusted results after controlling for confounding variables. Although this study was conducted prospectively, the collection of maternal urine and cord blood samples at the time of delivery and the assessment of fetal growth at that point limited our ability to establish causality between EDC exposure and fetal growth.

## Conclusion

BPA can impede fetal growth through various mechanisms, with particular attention given to those associated with placental insufficiency. Our study demonstrated an association between greater maternal BPA exposure and elevated fetal BPA levels, providing preliminary evidence of a link between BPA exposure and asymmetric fetal growth restriction due to placental insufficiency. Further research using large datasets is needed to strengthen these findings, paying particular attention to investigating the relationship between Doppler abnormalities and EDC exposure.

## Data availability statement

The datasets of the current study are available from the corresponding author on reasonable request.

## Ethics statement

The institutional review board of Seoul St. Mary’s Hospital approved the collection of the information for this study (XC21ONDI0125). The studies were conducted in accordance with the local legislation and institutional requirements. The participants provided their written informed consent to participate in this study.

## Author contributions

SH: Conceptualization, Data curation, Formal analysis, Investigation, Methodology, Resources, Writing – original draft. BK: Data curation, Resources, Writing – original draft. OK: Data curation, Resources, Writing – original draft. SW: Data curation, Resources, Writing – original draft. HSKi: Data curation, Resources, Writing – original draft. JW: Data curation, Resources, Writing – original draft. JS: Data curation, Resources, Writing – original draft. SC: Data curation, Resources, Writing – original draft. YJ: Data curation, Resources, Writing – original draft. YK: Data curation, Resources, Writing – original draft. MY: Data curation, Formal analysis, Writing – original draft. HWK: Data curation, Formal analysis, Writing – original draft. D-WL: Conceptualization, Investigation, Methodology, Writing – original draft. IP: Data curation, Resources, Writing – original draft. JP: Conceptualization, Investigation, Supervision, Validation, Writing – review & editing. HSKo: Conceptualization, Data curation, Funding acquisition, Resources, Supervision, Validation, Writing – review & editing.

## Footnotes

^1^ http://www.r-project.org

## References

[1] Ankit Kumar KA Jain V Deovanshi A Lepcha A Das C. Environmental impact of COVID-19 pandemic: more negatives than positives. Environ Sustain. (2021) 4:447–54. doi: 10.1007/s42398-021-00159-9 PMC7896832 38624614

[2] Behera JK Mishra P Jena AK Bhattacharya M Behera B. Understanding of environmental pollution and its anthropogenic impacts on biological resources during the COVID-19 period. Environ Sci Pollut Res Int. (2022) 29:1–16. doi: 10.1007/s11356-022-24789-6, PMID: 36580239 PMC9797902

[3] Prata JC Silva ALP Walker TR Duarte AC Rocha-Santos T. COVID-19 pandemic repercussions on the use and Management of Plastics. Environ Sci Technol. (2020) 54:7760–5. doi: 10.1021/acs.est.0c02178, PMID: 32531154

[4] Deierlein AL Grayon AR Zhu X Sun Y Liu X Kohlasch K. Personal care and household cleaning product use among pregnant women and new mothers during the COVID-19 pandemic. Int J Environ Res Public Health. (2022) 19:5645. doi: 10.3390/ijerph19095645, PMID: 35565038 PMC9104147

[5] Herbstman JB Romano ME Li X Jacobson LP Margolis AE Hamra GB. Characterizing changes in behaviors associated with chemical exposures during the COVID-19 pandemic. PLoS One. (2023) 18:e0277679. doi: 10.1371/journal.pone.0277679, PMID: 36638141 PMC9838870

[6] Ma Y Liu H Wu J Yuan L Wang Y Du X. The adverse health effects of bisphenol a and related toxicity mechanisms. Environ Res. (2019) 176:108575. doi: 10.1016/j.envres.2019.108575 31299621

[7] Özel F Rüegg J. Exposure to endocrine-disrupting chemicals and implications for neurodevelopment. Dev Med Child Neurol. (2023) 65:1005–11. doi: 10.1111/dmcn.15551, PMID: 36808586

[8] Patisaul HB. REPRODUCTIVE TOXICOLOGY: endocrine disruption and reproductive disorders: impacts on sexually dimorphic neuroendocrine pathways. Reproduction. (2021) 162:F111–30. doi: 10.1530/REP-20-0596, PMID: 33929341 PMC8484365

[9] Papalou O Kandaraki EA Papadakis G Diamanti-Kandarakis E. Endocrine disrupting chemicals: an occult mediator of metabolic disease. Front Endocrinol. (2019) 10:112. doi: 10.3389/fendo.2019.00112, PMID: 30881345 PMC6406073

[10] Modica R Benevento E Colao A. Endocrine-disrupting chemicals (EDCs) and cancer: new perspectives on an old relationship. J Endocrinol Investig. (2023) 46:667–77. doi: 10.1007/s40618-022-01983-4, PMID: 36526827

[11] Padmanabhan V Moeller J Puttabyatappa M. Impact of gestational exposure to endocrine disrupting chemicals on pregnancy and birth outcomes. Adv Pharmacol. (2021) 92:279–346. doi: 10.1016/bs.apha.2021.04.004, PMID: 34452689

[12] Haggerty DK Upson K Pacyga DC Franko JE Braun JM Strakovsky RS. REPRODUCTIVE TOXICOLOGY: pregnancy exposure to endocrine disrupting chemicals: implications for women’s health. Reproduction. (2021) 162:F169–80. doi: 10.1530/REP-21-0051, PMID: 34486984 PMC8511181

[13] Basak S Das MK Duttaroy AK. Plastics derived endocrine-disrupting compounds and their effects on early development. Birth Defects Res. (2020) 112:1308–25. doi: 10.1002/bdr2.1741, PMID: 32476245

[14] Mallozzi M Bordi G Garo C Caserta D. The effect of maternal exposure to endocrine disrupting chemicals on fetal and neonatal development: a review on the major concerns. Birth Defects Res C Embryo Today. (2016) 108:224–42. doi: 10.1002/bdrc.21137, PMID: 27653964

[15] Vrachnis N Loukas N Vrachnis D Antonakopoulos N Zygouris D Kοlialexi A. A systematic review of bisphenol a from dietary and non-dietary sources during pregnancy and its possible connection with fetal growth restriction: investigating its potential effects and the window of fetal vulnerability. Nutrients. (2021) 13:2426. doi: 10.3390/nu13072426, PMID: 34371934 PMC8308698

[16] Zhou Z Lei Y Wei W Zhao Y Jiang Y Wang N. Association between prenatal exposure to bisphenol a and birth outcomes: a systematic review with meta-analysis. Medicine (Baltimore). (2019) 98:e17672. doi: 10.1097/MD.0000000000017672, PMID: 31689782 PMC6946218

[17] Hu C-Y Li F-L Hua X-G Jiang W Mao C Zhang X-J. The association between prenatal bisphenol a exposure and birth weight: a meta-analysis. Reprod Toxicol. (2018) 79:21–31. doi: 10.1016/j.reprotox.2018.04.013, PMID: 29709518

[18] DiVall SA. The influence of endocrine disruptors on growth and development of children. Curr Opin Endocrinol Diabetes Obes. (2013) 20:50–5. doi: 10.1097/MED.0b013e32835b7ee6 23222850

[19] Kamai EM McElrath TF Ferguson KK. Fetal growth in environmental epidemiology: mechanisms, limitations, and a review of associations with biomarkers of non-persistent chemical exposures during pregnancy. Environ Health. (2019) 18:43. doi: 10.1186/s12940-019-0480-8, PMID: 31068204 PMC6505101

[20] Latini G Del Vecchio A Massaro M Verrotti A De Felice C. In utero exposure to phthalates and fetal development. Curr Med Chem. (2006) 13:2527–34. doi: 10.2174/092986706778201666 17017909

[21] Gui S-Y Chen Y-N Wu K-J Liu W Wang W-J Liang H-R. Association between exposure to per-and polyfluoroalkyl substances and birth outcomes: a systematic review and meta-analysis. Front Public Health. (2022) 10:855348. doi: 10.3389/fpubh.2022.855348, PMID: 35400049 PMC8988915

[22] Negri E Metruccio F Guercio V Tosti L Benfenati E Bonzi R. Exposure to PFOA and PFOS and fetal growth: a critical merging of toxicological and epidemiological data. Crit Rev Toxicol. (2017) 47:489–515. doi: 10.1080/10408444.2016.1271972, PMID: 28617200

[23] Bach CC Bech BH Brix N Nohr EA Bonde JPE Henriksen TB. Perfluoroalkyl and polyfluoroalkyl substances and human fetal growth: a systematic review. Crit Rev Toxicol. (2015) 45:53–67. doi: 10.3109/10408444.2014.952400, PMID: 25372700

[24] Johnson PI Sutton P Atchley DS Koustas E Lam J Sen S. The navigation guide—evidence-based medicine meets environmental health: systematic review of human evidence for PFOA effects on fetal growth. Environ Health Perspect. (2014) 122:1028–39. doi: 10.1289/ehp.1307893, PMID: 24968388 PMC4181929

[25] Pels A Beune IM van Wassenaer-Leemhuis AG Limpens J Ganzevoort W. Early-onset fetal growth restriction: a systematic review on mortality and morbidity. Acta Obstet Gynecol Scand. (2020) 99:153–66. doi: 10.1111/aogs.13702, PMID: 31376293 PMC7004054

[26] Garite TJ Clark R Thorp JA. Intrauterine growth restriction increases morbidity and mortality among premature neonates. Am J Obstet Gynecol. (2004) 191:481–7. doi: 10.1016/j.ajog.2004.01.036 15343225

[27] Malhotra A Allison BJ Castillo-Melendez M Jenkin G Polglase GR Miller SL. Neonatal morbidities of fetal growth restriction: pathophysiology and impact. Front Endocrinol. (2019) 10:55. doi: 10.3389/fendo.2019.00055, PMID: 30792696 PMC6374308

[28] Mifsud W Sebire NJ. Placental pathology in early-onset and late-onset fetal growth restriction. Fetal Diagn Ther. (2014) 36:117–28. doi: 10.1159/000359969 24577279

[29] Hiersch L Melamed N. Fetal growth velocity and body proportion in the assessment of growth. Am J Obstet Gynecol. (2018) 218:S700–S711.e1. doi: 10.1016/j.ajog.2017.12.014, PMID: 29422209

[30] Hershkovitz R Kingdom JC Geary M Rodeck CH. Fetal cerebral blood flow redistribution in late gestation: identification of compromise in small fetuses with normal umbilical artery Doppler. Ultrasound Obstet Gynecol. (2000) 15:209–12. doi: 10.1046/j.1469-0705.2000.00079.x, PMID: 10846776

[31] Dashe JS McIntire DD Lucas MJ Leveno KJ. Effects of symmetric and asymmetric fetal growth on pregnancy outcomes. Obstet Gynecol. (2000) 96:321–7. doi: 10.1016/s0029-7844(00)00943-1, PMID: 10960619

[32] David C Gabrielli S Pilu G Bovicelli L. The head-to-abdomen circumference ratio: a reappraisal. Ultrasound Obstet Gynecol. (1995) 5:256–9. doi: 10.1046/j.1469-0705.1995.05040256.x, PMID: 7600207

[33] Street ME Bernasconi S. Endocrine-disrupting chemicals in human fetal growth. Int J Mol Sci. (2020) 21:1430. doi: 10.3390/ijms21041430, PMID: 32093249 PMC7073082

[34] Yang C Song G Lim W. A mechanism for the effect of endocrine disrupting chemicals on placentation. Chemosphere. (2019) 231:326–36. doi: 10.1016/j.chemosphere.2019.05.133 31132539

[35] Gingrich J Ticiani E Veiga-Lopez A. Placenta disrupted: endocrine disrupting chemicals and pregnancy. Trends Endocrinol Metab. (2020) 31:508–24. doi: 10.1016/j.tem.2020.03.003, PMID: 32249015 PMC7395962

[36] Müller JE Meyer N Santamaria CG Schumacher A Luque EH Zenclussen ML. Bisphenol a exposure during early pregnancy impairs uterine spiral artery remodeling and provokes intrauterine growth restriction in mice. Sci Rep. (2018) 8:9196. doi: 10.1038/s41598-018-27575-y, PMID: 29907759 PMC6003928

[37] Barberio L Paulesu L Canesi L Grasselli E Mandalà M. Bisphenol a interferes with uterine artery features and impairs rat feto-placental growth. Int J Mol Sci. (2021) 22:6912. doi: 10.3390/ijms22136912, PMID: 34199136 PMC8268965

[38] Tachibana T Wakimoto Y Nakamuta N Phichitraslip T Wakitani S Kusakabe K. Effects of bisphenol a (BPA) on placentation and survival of the neonates in mice. J Reprod Dev. (2007) 53:509–14. doi: 10.1262/jrd.18171, PMID: 17384489

[39] Lan X Fu L-J Zhang J Liu X-Q Zhang H-J Zhang X. Bisphenol a exposure promotes HTR-8/SVneo cell migration and impairs mouse placentation involving upregulation of integrin-β1 and MMP-9 and stimulation of MAPK and PI3K signaling pathways. Oncotarget. (2017) 8:51507–21. doi: 10.18632/oncotarget.17882, PMID: 28881663 PMC5584264

[40] Yang M Ryu J-H Jeon R Kang D Yoo K-Y. Effects of bisphenol a on breast cancer and its risk factors. Arch Toxicol. (2009) 83:281–5. doi: 10.1007/s00204-008-0364-0 18843480

[41] Papageorghiou AT Ohuma EO Altman DG Todros T Ismail LC Lambert A. International standards for fetal growth based on serial ultrasound measurements: the fetal growth longitudinal study of the INTERGROWTH-21st project. Lancet. (2014) 384:869–79. doi: 10.1016/S0140-6736(14)61490-2, PMID: 25209488

[42] Stirnemann J Villar J Salomon LJ Ohuma E Ruyan P Altman DG. International estimated fetal weight standards of the INTERGROWTH-21st project. Ultrasound Obstet Gynecol. (2017) 49:478–86. doi: 10.1002/uog.17347, PMID: 27804212 PMC5516164

[43] Campbell S Thoms A. Ultrasound measurement of the fetal head to abdomen circumference ratio in the assessment of growth retardation. Br J Obstet Gynaecol. (1977) 84:165–74. doi: 10.1111/j.1471-0528.1977.tb12550.x, PMID: 843490

[44] Quinton A Cook C Peek M. The prediction of the small for gestational age fetus with the head circumference to abdominal circumference (HC/AC) ratio: a new look at an old measurement. Sonography. (2015) 2:27–31. doi: 10.1002/sono.12022

[45] Society for Maternal-Fetal Medicine (SMFM) Martins JG Biggio JR Abuhamad A. Society for maternal-fetal medicine consult series# 52: diagnosis and management of fetal growth restriction:(replaces clinical guideline number 3, April 2012). Am J Obstet Gynecol. (2020) 223:B2–B17. doi: 10.1016/j.ajog.2020.05.010 32407785

[46] Pedhazur EJ Schmelkin LP. Measurement, design, and analysis: An integrated approach. Hove: Psychology Press (2013).

[47] Zbucka-Krętowska M Łazarek U Miltyk W Sidorkiewicz I Pierzyński P Milewski R. Simultaneous analysis of bisphenol a fractions in maternal and fetal compartments in early second trimester of pregnancy. J Perinat Med. (2019) 47:765–70. doi: 10.1515/jpm-2019-0040, PMID: 31348763

[48] Pinney SE Mesaros CA Snyder NW Busch CM Xiao R Aijaz S. Second trimester amniotic fluid bisphenol a concentration is associated with decreased birth weight in term infants. Reprod Toxicol. (2017) 67:1–9. doi: 10.1016/j.reprotox.2016.11.007, PMID: 27829162 PMC5303174

[49] Chou W-C Chen J-L Lin C-F Chen Y-C Shih F-C Chuang C-Y. Biomonitoring of bisphenol a concentrations in maternal and umbilical cord blood in regard to birth outcomes and adipokine expression: a birth cohort study in Taiwan. Environ Health. (2011) 10:94. doi: 10.1186/1476-069X-10-94, PMID: 22050967 PMC3225308

[50] Troisi J Mikelson C Richards S Symes S Adair D Zullo F. Placental concentrations of bisphenol a and birth weight from births in the southeastern US. Placenta. (2014) 35:947–52. doi: 10.1016/j.placenta.2014.08.091, PMID: 25227326

[51] Huo W Xia W Wan Y Zhang B Zhou A Zhang Y. Maternal urinary bisphenol a levels and infant low birth weight: a nested case–control study of the health baby cohort in China. Environ Int. (2015) 85:96–103. doi: 10.1016/j.envint.2015.09.005, PMID: 26382648

[52] Huang Y-F Pan W-C Tsai Y-A Chang C-H Chen P-J Shao Y-s. Concurrent exposures to nonylphenol, bisphenol a, phthalates, and organophosphate pesticides on birth outcomes: a cohort study in Taipei, Taiwan. Sci Total Environ. (2017) 607-608:1126–35. doi: 10.1016/j.scitotenv.2017.07.092, PMID: 28724251

[53] Casas M Valvi D Ballesteros-Gomez A Gascon M Fernández MF Garcia-Esteban R. Exposure to bisphenol a and phthalates during pregnancy and ultrasound measures of fetal growth in the INMA-Sabadell cohort. Environ Health Perspect. (2016) 124:521–8. doi: 10.1289/ehp.1409190, PMID: 26196298 PMC4829997

[54] Snijder CA Heederik D Pierik FH Hofman A Jaddoe VW Koch HM. Fetal growth and prenatal exposure to bisphenol a: the generation R study. Environ Health Perspect. (2013) 121:393–8. doi: 10.1289/ehp.1205296, PMID: 23459363 PMC3621207

[55] Amin MM Ghasemi Z Khoshhali M Taheri E Dehdashti B Fatehizadeh A. Association of maternal exposure to bisphenol a with her β-hCG level and neonatal anthropometric measures. Environ Sci Pollut Res Int. (2021) 28:62809–15. doi: 10.1007/s11356-021-15094-9, PMID: 34215981

[56] Smarr MM Grantz KL Sundaram R Maisog JM Kannan K Louis GMB. Parental urinary biomarkers of preconception exposure to bisphenol a and phthalates in relation to birth outcomes. Environ Health. (2015) 14:73. doi: 10.1186/s12940-015-0060-5, PMID: 26362861 PMC4567813

[57] Yang P Lin B-G Zhou B Cao W-C Chen P-P Deng Y-L. Sex-specific associations of prenatal exposure to bisphenol a and its alternatives with fetal growth parameters and gestational age. Environ Int. (2021) 146:106305. doi: 10.1016/j.envint.2020.106305, PMID: 33395947

[58] Lee B-E Park H Hong Y-C Ha M Kim Y Chang N. Prenatal bisphenol a and birth outcomes: MOCEH (mothers and Children's environmental health) study. Int J Hyg Environ Health. (2014) 217:328–34. doi: 10.1016/j.ijheh.2013.07.005, PMID: 23911140

[59] Streja E Miller JE Wu C Bech BH Pedersen LH Schendel DE. Disproportionate fetal growth and the risk for congenital cerebral palsy in singleton births. PLoS One. (2015) 10:e0126743. doi: 10.1371/journal.pone.0126743, PMID: 25974407 PMC4431832

[60] Uldbjerg CS Lim Y-H Krause M Frederiksen H Andersson A-M Bräuner EV. Sex-specific associations between maternal exposure to parabens, phenols and phthalates during pregnancy and birth size outcomes in offspring. Sci Total Environ. (2022) 836:155565. doi: 10.1016/j.scitotenv.2022.155565, PMID: 35508231

[61] Philippat C Botton J Calafat AM Ye X Charles M-A Slama R. Prenatal exposure to phenols and growth in boys. Epidemiology. (2014) 25:625–35. doi: 10.1097/EDE.0000000000000132, PMID: 25061923 PMC4724208

[62] Goodrich JM Ingle ME Domino SE Treadwell MC Dolinoy DC Burant C. First trimester maternal exposures to endocrine disrupting chemicals and metals and fetal size in the Michigan mother–infant pairs study. J Dev Orig Health Dis. (2019) 10:447–58. doi: 10.1017/S204017441800106X, PMID: 30696509 PMC6660406

[63] McMillen IC Adams MB Ross JT Coulter CL Simonetta G Owens JA. Fetal growth restriction: adaptations and consequences. Reproduction. (2001) 122:195–204. doi: 10.1530/rep.0.1220195 11467970

[64] Giussani DA. The fetal brain sparing response to hypoxia: physiological mechanisms. J Physiol. (2016) 594:1215–30. doi: 10.1113/JP271099, PMID: 26496004 PMC4721497

[65] Spagnoletti A Paulesu L Mannelli C Ermini L Romagnoli R Cintorino M. Low concentrations of bisphenol a and Para-Nonylphenol affect extravillous pathway of human trophoblast cells. Mol Cell Endocrinol. (2015) 412:56–64. doi: 10.1016/j.mce.2015.05.023, PMID: 26027920

[66] Song W Puttabyatappa M Zeng L Vazquez D Pennathur S Padmanabhan V. Developmental programming: prenatal bisphenol a treatment disrupts mediators of placental function in sheep. Chemosphere. (2020) 243:125301. doi: 10.1016/j.chemosphere.2019.125301, PMID: 31726260 PMC7243413

[67] Ponniah M Billett EE De Girolamo LA. Bisphenol a increases BeWo trophoblast survival in stress-induced paradigms through regulation of oxidative stress and apoptosis. Chem Res Toxicol. (2015) 28:1693–703. doi: 10.1021/acs.chemrestox.5b00093, PMID: 26247420

[68] Elmetwally MA Halawa AA Tang W Wu G Bazer FW. Effects of bisphenol a on expression of genes related to amino acid transporters, insulin-like growth factor, aquaporin and amino acid release by porcine trophectoderm cells. Reprod Toxicol. (2020) 96:241–8. doi: 10.1016/j.reprotox.2020.07.008, PMID: 32710935

[69] Ye Y Tang Y Xiong Y Feng L Li X. Bisphenol a exposure alters placentation and causes preeclampsia-like features in pregnant mice involved in reprogramming of DNA methylation of WNT2. FASEB J. (2019) 33:2732–42. doi: 10.1096/fj.201800934RRR, PMID: 30303745 PMC7021011

[70] Ferguson KK McElrath TF Cantonwine DE Mukherjee B Meeker JD. Phthalate metabolites and bisphenol-a in association with circulating angiogenic biomarkers across pregnancy. Placenta. (2015) 36:699–703. doi: 10.1016/j.placenta.2015.04.002, PMID: 25913709 PMC4441857

[71] Bloom MS Wenzel AG Brock JW Kucklick JR Wineland RJ Cruze L. Racial disparity in maternal phthalates exposure; association with racial disparity in fetal growth and birth outcomes. Environ Int. (2019) 127:473–86. doi: 10.1016/j.envint.2019.04.005, PMID: 30981018

[72] Messerlian C Braun JM Mínguez-Alarcón L Williams PL Ford JB Mustieles V. Paternal and maternal urinary phthalate metabolite concentrations and birth weight of singletons conceived by subfertile couples. Environ Int. (2017) 107:55–64. doi: 10.1016/j.envint.2017.06.015, PMID: 28666241 PMC5563279

[73] Costa O Iñiguez C Manzano-Salgado CB Amiano P Murcia M Casas M. First-trimester maternal concentrations of polyfluoroalkyl substances and fetal growth throughout pregnancy. Environ Int. (2019) 130:104830. doi: 10.1016/j.envint.2019.05.024, PMID: 31247476

[74] Meng Q Inoue K Ritz B Olsen J Liew Z. Prenatal exposure to perfluoroalkyl substances and birth outcomes; an updated analysis from the Danish National Birth Cohort. Int J Environ Res Public Health. (2018) 15:1832. doi: 10.3390/ijerph15091832, PMID: 30149566 PMC6164159

[75] Lauritzen HB Larose TL Øien T Sandanger TM Odland JØ Van De Bor M. Maternal serum levels of perfluoroalkyl substances and organochlorines and indices of fetal growth: a Scandinavian case–cohort study. Pediatr Res. (2017) 81:33–42. doi: 10.1038/pr.2016.187, PMID: 27656770 PMC5313514

[76] Lee YJ Kim M-K Bae J Yang J-H. Concentrations of perfluoroalkyl compounds in maternal and umbilical cord sera and birth outcomes in Korea. Chemosphere. (2013) 90:1603–9. doi: 10.1016/j.chemosphere.2012.08.035, PMID: 22990023

[77] Santos S Sol CM Van Zwol-Janssens C Philips EM Asimakopoulos AG Martinez-Moral M-P. Maternal phthalate urine concentrations, fetal growth and adverse birth outcomes. A population-based prospective cohort study. Environ Int. (2021) 151:106443. doi: 10.1016/j.envint.2021.106443, PMID: 33610054

[78] Gao H Xu Y-y Huang K Ge X Zhang Y-w Yao H-y. Cumulative risk assessment of phthalates associated with birth outcomes in pregnant Chinese women: a prospective cohort study. Environ Pollut. (2017) 222:549–56. doi: 10.1016/j.envpol.2016.11.026 28024814

[79] Kalloo G Wellenius GA McCandless L Calafat AM Sjodin A Romano ME. Exposures to chemical mixtures during pregnancy and neonatal outcomes: the HOME study. Environ Int. (2020) 134:105219. doi: 10.1016/j.envint.2019.105219, PMID: 31726361

[80] Hjermitslev MH Long M Wielsøe M Bonefeld-Jørgensen EC. Persistent organic pollutants in Greenlandic pregnant women and indices of foetal growth: the ACCEPT study. Sci Total Environ. (2020) 698:134118. doi: 10.1016/j.scitotenv.2019.134118, PMID: 31494415

[81] Wang H Du H Yang J Jiang H Karmin O Xu L. PFOS, PFOA, estrogen homeostasis, and birth size in Chinese infants. Chemosphere. (2019) 221:349–55. doi: 10.1016/j.chemosphere.2019.01.061, PMID: 30641376

[82] Lee YJ Ryu H-Y Kim H-K Min CS Lee JH Kim E. Maternal and fetal exposure to bisphenol a in Korea. Reprod Toxicol. (2008) 25:413–9. doi: 10.1016/j.reprotox.2008.05.058, PMID: 18577445

[83] Lee J Choi K Park J Moon H-B Choi G Lee JJ. Bisphenol a distribution in serum, urine, placenta, breast milk, and umbilical cord serum in a birth panel of mother–neonate pairs. Sci Total Environ. (2018) 626:1494–501. doi: 10.1016/j.scitotenv.2017.10.042, PMID: 29146078

[84] Kim S Park E Park E-K Lee S Kwon J-A Shin B-H. Urinary concentrations of bisphenol mixtures during pregnancy and birth outcomes: the MAKE study. Int J Environ Res Public Health. (2021) 18:10098. doi: 10.3390/ijerph181910098, PMID: 34639400 PMC8508042

[85] Lee G Kim S Kho Y Kim S Lee S Choi G. Urinary levels of phthalates and DINCH metabolites in Korean and Thai pregnant women across three trimesters. Sci Total Environ. (2020) 711:134822. doi: 10.1016/j.scitotenv.2019.134822, PMID: 31818591

[86] Kim S Eom S Kim H-J Lee JJ Choi G Choi S. Association between maternal exposure to major phthalates, heavy metals, and persistent organic pollutants, and the neurodevelopmental performances of their children at 1 to 2 years of age-CHECK cohort study. Sci Total Environ. (2018) 624:377–84. doi: 10.1016/j.scitotenv.2017.12.058, PMID: 29258038

[87] Mørck TJ Sorda G Bechi N Rasmussen BS Nielsen JB Ietta F. Placental transport and in vitro effects of bisphenol a. Reprod Toxicol. (2010) 30:131–7. doi: 10.1016/j.reprotox.2010.02.007, PMID: 20214975

[88] Balakrishnan B Henare K Thorstensen EB Ponnampalam AP Mitchell MD. Transfer of bisphenol a across the human placenta. Am J Obstet Gynecol. (2010) 202:393.e1–7. doi: 10.1016/j.ajog.2010.01.025, PMID: 20350650

[89] Mose T Mortensen GK Hedegaard M Knudsen LE. Phthalate monoesters in perfusate from a dual placenta perfusion system, the placenta tissue and umbilical cord blood. Reprod Toxicol. (2007) 23:83–91. doi: 10.1016/j.reprotox.2006.08.006, PMID: 17049806

[90] Maekawa R Ito R Iwasaki Y Saito K Akutsu K Takatori S. Evidence of exposure to chemicals and heavy metals during pregnancy in Japanese women. Reprod Med Biol. (2017) 16:337–48. doi: 10.1002/rmb2.12049, PMID: 29259487 PMC5715897

