# Supplementary material for: The associations between maternal and fetal exposure to endocrine-disrupting chemicals and asymmetric fetal growth restriction: a prospective cohort study
Source: Front Public Health. 2024 Apr 11;12:1351786. doi: 10.3389/fpubh.2024.1351786 (PMC11043493; doi:10.3389/fpubh.2024.1351786)
Supplement: Supplementary file 1 [file Data_Sheet_1.docx]

**Supplementary Methods**

**Analyses of BPA, MEP and PFOA**

We analyzed BPA and MEP in serum and urine, following Yi et al.^1^ and Frederiksen et al.’s^2^ methods, respectively, with minor modification. The column was Agilent Zorbax Eclipse Plus C18 column (2.1 x 50mm, 1.8 micron). The column temperature was maintained at 40°C. The mobile phase was a binary mixture of 5 mM of ammonium acetate in water (A) and methanol (B). These two mobile phases were used in a gradient mode at a flow rate of 0.3 mL/min. The gradient of B was increased from 20 % to 100 % over 10 min with a linear gradient, retained 100 % for 2 min and switched to 20% for 0.5 min. Ten µL of each sample was injected into the UHPLC system. For MS, the electric parameters were set up as negative mode, 3,500 V capillary voltage of the EI source, 1,500 V nozzle voltage**.** For drying liquids from UHPLC, nebulizer gas flow and sheath gas flow were 10 L/min. The temperatures of nebulizer and sheath gases were 250 °C and 300 °C, respectively. The collision energy was 15V for BPA and 17V for MEP.

We also quantified serum and urinary PFOA, following Liu et al.^3^ and Worley et al.^4^ or Yao et al.’s^5^ methods, respectively. The column of the UHPLC was ACME C18 UHPLC column (2.1 x 50mm, 1.9 micron). The column temperature was maintained at 40◦C. The mobile phase was a binary mixture of 2 mM ammonium acetate in water (A) and methanol (B) at a flow rate of 0.3 mL/min. The gradient of B was increased from 5% to 95% over 3 min, retained 95% for 0.5 min, and switched to 5% of for 0.2 min. Two µL of each sample was injected into the UHPLC-MS/MS system. For MS, Bruker EVOQ Qube LC-Triple Quadrupole (Bruker corporation, Billerica, MA, USA) was used with heated ESI (HESI) interface. The electric parameters were set up as negative mode, 3500 V capillary voltage of the EI source. The nebulizer gas flow, cone gas flow, and probe gas flow were 60L/min, 20L/min, and 40L/min, respectively. The cone temperature and heated probe temperature were 250◦C, respectively. The collision energy and collision gas were 3eV and argon gas, respectively.

Urinary creatinine was analyzed with an automatic biomedical analyzer (HITACHI 7020, Tokyo, Japan) to adjust the urine density.


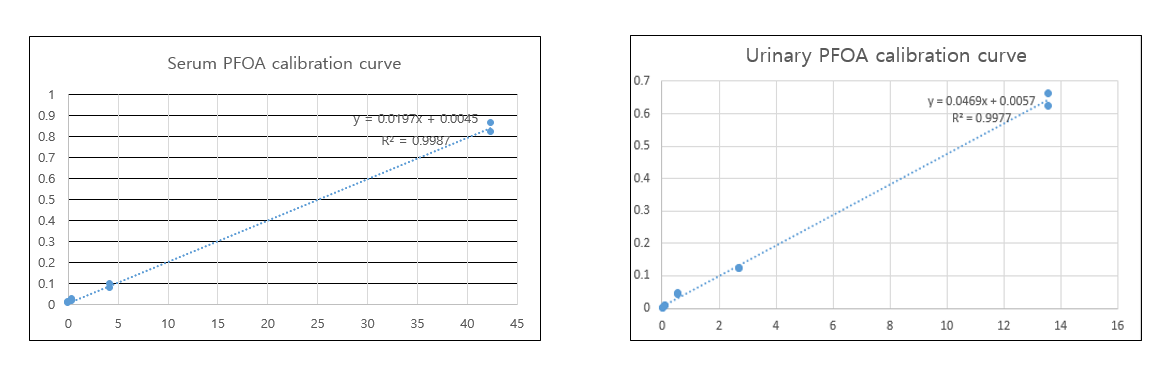


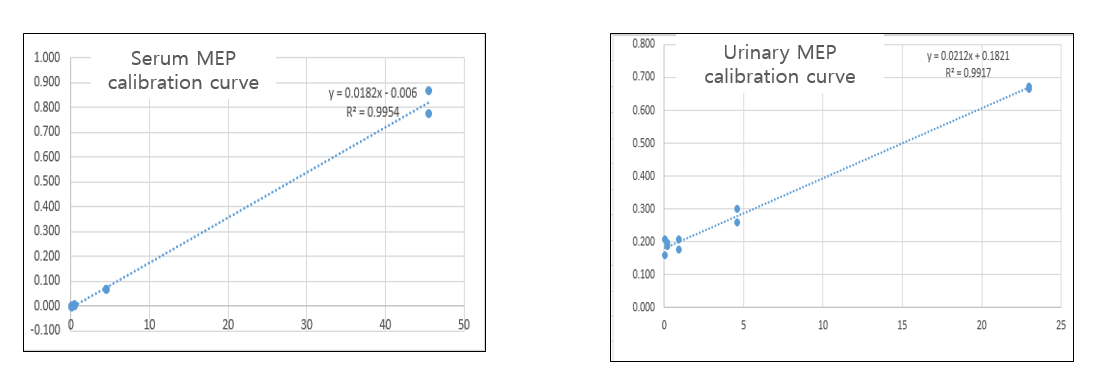


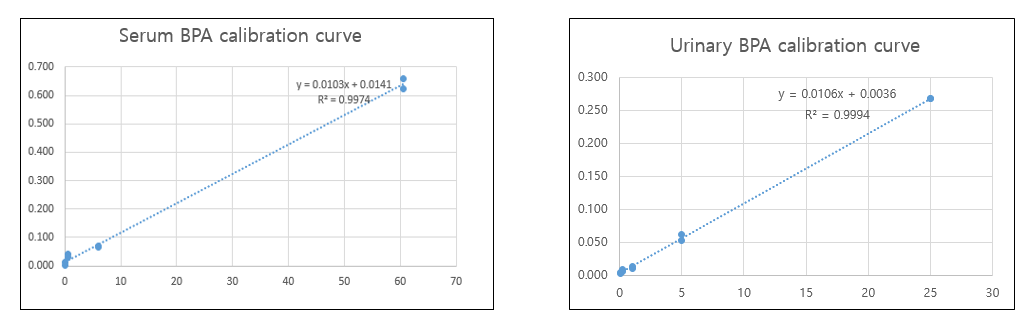


**REFERENCES**

1. Yi B, Kim C, Yang M. Biological monitoring of bisphenol A with HLPC/FLD and LC/MS/MS assays. J Chromatogr B 2010;878:2606-2610.

2. Frederiksen H, Jørgensen N, & Andersson AM. Correlations between phthalate metabolites in urine, serum, and seminal plasma from young Danish men determined by isotope dilution liquid chromatography tandem mass spectrometry. J Anal Toxicol 2010:34:400-410.

3. Liu WS, Lai YT, Chan HL, Li SY, Lin CC, Liu CK et al. Associations between perfluorinated chemicals and serum biochemical markers and performance status in uremic patients under hemodialysis. PloS One 2018;13(7):e0200271.

4. Worley RR, Moore SM, Tierney BC, Ye X, Calafat AM, Campbell S et al. Per-and polyfluoroalkyl substances in human serum and urine samples from a residentially exposed community. Environ Int 2017;106:135-143.

5. Yao J, Pan Y, Huan Y, Dai J. Occurrence of Novel Perfluoroalkyl Ether Carboxylic Acids in River Water and Human Urine Quantified by a Simple Liquid–Liquid Microextraction Approach Coupled with LC–MS/MS. Environmental Science & Technology Letters, 2021;8(9), 773-778.
