# Supplementary material for: The associations between maternal and fetal exposure to endocrine-disrupting chemicals and asymmetric fetal growth restriction: a prospective cohort study
Source: Front Public Health. 2024 Apr 11;12:1351786. doi: 10.3389/fpubh.2024.1351786 (PMC11043493; doi:10.3389/fpubh.2024.1351786)
Supplement: Supplementary file 2 [file Data_Sheet_2.docx]

**Table S1** Distribution of the bisphenol-A, monoethyl phthalate, and perfluorooctanoic acid in maternal urine and fetal cord blood.

|  |  |  |  |  | Percentile | | | | | |
| --- | --- | --- | --- | --- | --- | --- | --- | --- | --- | --- |
|  |  | N(%) >LOD | GM | Min | 10th | 25^th^ | 50^th^ | 75^th^ | 90^th^ | Max |
| BPA | Maternal urine (ug/g cre) | 136 (93.2) | 1.220 | ND | 0.107 | 0.755 | 1.812 | 3.765 | 8.398 | 30.518 |
|  | Fetal cord blood (ug/L) | 133 (91.1) | 0.751 | ND | 0.010 | 0.290 | 0.859 | 1.683 | 6.065 | 19.679 |
| MEP | Maternal urine (ug/g cre) | 133 (91.1) | 10.523 | ND | 0.369 | 5.303 | 15.195 | 38.370 | 95.598 | 1284.840 |
|  | Fetal cord blood (ug/L) | 113 (77.4) | 0.106 | ND | ND | 0.010 | 0.260 | 0.654 | 2.129 | 7.040 |
| PFOA | Maternal urine (ug/g cre) | 72 (49.3) | 0.026 | ND | ND | ND | ND | 0.123 | 0.349 | 7.450 |
|  | Fetal cord blood (ug/L) | 146 (100) | 2.315 | 0.321 | 0.950 | 1.486 | 2.450 | 3.936 | 4.987 | 8.827 |

Abbreviations: LOD, limit of detection; GM, geometric mean; BPA, bisphenol-A; MEP, monoethyl phthalate; PFOA, perfluorooctanoic acid.

**TABLE S2** Baseline characteristics between Non-FGR group and FGR group, or between non FGR group and FGR with asymmetry group.

|  | Non-FGR  (n=129) | FGR  (n=17) | *P* value^a^ | FGR with asymmetry (n=9) | *P* value^b^ |
| --- | --- | --- | --- | --- | --- |
| Maternal age (years) | 34 (32, 38) | 35 (30, 39) | 0.569 | 35 (31, 41) | 0.997 |
| Age ≥35 | 64 (49.6) | 9 (52.9) | 0.796 | 5 (55.6) | 1.000 |
| Nulliparity | 75 (58.1) | 11 (64.7) | 0.605 | 6 (66.7) | 0.736 |
| Pre-pregnancy BMI (kg/m^2^) | 21.2 (19.5, 23.8) | 20.4 (18.9, 22.7) | 0.180 | 20.4 (19.2, 22.3) | 0.283 |
| BMI ≥23 | 39 (30.2) | 3 (17.6) | 0.396 | 0 (0.0) | 0.061 |
| Pre-pregnancy smoking | **7 (5.4)** | **4 (23.5)** | **0.025** | 2 (22.2) | 0.107 |
| Pre-pregnancy alcohol consumption | 83 (64.3) | 14 (82.4) | 0.139 | 7 (77.8) | 0.496 |
| Preexisting diabetes mellitus | 0 (0.0) | 0 (0.0) | - | 0 (0.0) | - |
| Preexisting hypertension | 5 (3.9) | 1 (5.9) | 0.531 | 0 (0.0) | 1.000 |
| Gestational diabetes mellitus | 38 (29.5) | 2 (11.8) | 0.156 | 0 (0.0) | 0.063 |
| Pregnancy associated hypertension | 4 (3.1) | 1 (5.9) | 0.466 | 0 (0.0) | 1.000 |
| GA at ultrasound exam (weeks) | 38.3 (37.7, 39.1) | 38.9 (37.9, 39.9) | 0.184 | 38.4 (37.6, 39.5) | 0.766 |
| EFW (g) | **3147 (2940, 3400)** | **2814 (2518, 3044)** | **<0.001** | **2811 (2366, 3109)** | **0.004** |
| GA at delivery (weeks) | 38.6 (38.0, 39.5) | 38.9 (38.1, 40.0) | 0.285 | 38.7 (38.0, 39.6) | 0.863 |
| Cesarean delivery | 72 (55.8) | 13 (76.5) | 0.105 | 7 (77.8) | 0.300 |
| Sex |  |  | 0.355 |  | 0.738 |
| Boy | 53 (41.1) | 5 (29.4) |  | 3 (33.3) |  |
| Girls | 76 (58.9) | 12 (70.6) |  | 6 (66.7) |  |
| NICU admission | 18 (14.0) | 1 (5.9) | 0.700 | 0 (0.0) | 0.605 |
| Birthweight (g) | **3135 (2885, 3420)** | **2790 (2493, 3088)** | **0.001** | **2790 (2550, 3130)** | **0.031** |

^a^Comparison between FGR group and Non-FGR group.

^b^Comparison between FGR with asymmetry group and Non-FGR group.

FGR, fetal growth restriction; BMI, body mass index; GA, gestational age; EFW, estimated birth weight; NICU, neonatal intensive unit

**TABLE S3** Linear associations between EDCs and fetal growth indices using a multivariable linear regression model.

|  | BPD z-score |  | HC z-score |  | AC z-score |  | FL z-score |  | EFW z- score |  |
| --- | --- | --- | --- | --- | --- | --- | --- | --- | --- | --- |
|  | *β* | 95% CI | *β* | 95% CI | *β* | 95% CI | *β* | 95% CI | *β* | 95% CI |
| Maternal BPA | 0.0063 | -0.0577, 0.0702 | 0.0564 | -0.0134,  0.1261 | -0.0063 | -0.0596, 0.0469 | -0.0072 | -0.0764, 0.0620 | -0.0045 | -0.0512, 0.0423 |
| Fetal BPA | -0.0084 | -0.0624, 0.0455 | 0.0305 | -0.0286, 0.0896 | -0.0275 | -0.0721, 0.0172 | 0.0228 | -0.0355, 0.0810 | -0.0086 | -0.0480, 0.0308 |
| Maternal MEP | -0.0148 | -0.0667, 0.0371 | 0.0134 | -0.0437, 0.0705 | 0.0093 | -0.0339, 0.0525 | **0.0672** | **0.0121, 0.1223** | 0.0290 | -0.0086, 0.0667 |
| Fetal MEP | -0.0262 | -0.0747, 0.0224 | 0.0135 | -0.0401, 0.0670 | 0.0046 | -0.0360, 0.0451 | 0.0090 | -0.0437, 0.0617 | -0.0084 | -0.0439, 0.0272 |
| Maternal PFOA | 0.0185 | -0.0410, 0.0779 | 0.0045 | -0.0609, 0.0699 | 0.0086 | -0.0409, 0.0581 | -0.0267 | -0.0910, 0.0375 | -0.0080 | -0.0515, 0.0355 |
| Fetal PFOA | -0.1565 | -0.3290, 0.0159 | -0.1538 | -0.3439, 0.0363 | 0.0318 | -0.1133, 0.1768 | -0.0763 | -0.2646, 0.1120 | 0.0057 | -0.1332, 0.1218 |

The bold font means remained statistically significance after adjusting for maternal age, BMI, past smoker, GA at exam, and fetal sex. EDC, endocrine disrupting chemicals; BPD, biparietal diameter; HC, head circumference, AC; abdominal circumference; FL, femur length; EFW, estimated fetal weight; CI, confidence interval; BPA, bisphenol-A; MEP, monoethyl phthalate; PFOA, perfluorooctanoic acid.

**TABLE S4** The non-linear associations between exposure to EDCs and the growth parameters using GAM after adjusting for confounding factors

|  | BPD z-score |  |  | HC z-score |  |  | AC z-score |  |  |
| --- | --- | --- | --- | --- | --- | --- | --- | --- | --- |
|  | Edf | R^2^ | DE(%) | Edf | R^2^ | DE(%) | Edf | R^2^ | DE(%) |
| Maternal BPA | 1 | -0.0249 | 1.75 | 1 | 0.0279 | 6.81 | 1.686 | 0.0721 | 11.5 |
| Fetal BPA | 1 | -0.0245 | 1.79 | 1 | 0.0174 | 5.8 | 1 | 0.0702 | 10.9 |
| Maternal MEP | 1 | -0.0228 | 1.95 | 1 | 0.0115 | 5.24 | 1 | 0.0615 | 10 |
| Fetal MEP | 2.828 | -0.0014 | 5.27 | 1 | 0.0118 | 5.26 | 1 | 0.0606 | 9.95 |
| Maternal PFOA | 1 | -0.0224 | 1.99 | 1 | 0.0101 | 5.11 | 4.546 | 0.0973 | 15.7 |
| Fetal PFOA | 1.651 | 0.0089 | 5.43 | 1 | 0.0279 | 6.81 | **6.545** | **0.167** | **23.3** |
|  | FL z-score |  |  | EFW z score |  |  | HC/AC ratio |  |  |
|  | Edf | R^2^ | DE(%) | Edf | R^2^ | DE(%) | Edf | R^2^ | DE(%) |
| Maternal BPA | 4.984 | 0.0684 | 13.3 | 1 | 0.0576 | 9.66 | 1.616 | 0.0808 | 12.3 |
| Fetal BPA | 2.927 | 0.0448 | 9.7 | 1.769 | 0.0733 | 11.7 | **1** | **0.0821** | **12** |
| Maternal MEP | 6.432 | 0.0952 | 16.7 | 1 | 0.0728 | 11.1 | 1 | 0.0547 | 9.39 |
| Fetal MEP | 1 | 0.0167 | 5.74 | 3.163 | 0.0885 | 14 | 1 | 0.0552 | 9.43 |
| Maternal PFOA | 3.115 | 0.0418 | 9.54 | 4.588 | 0.114 | 17.2 | 1 | 0.0548 | 9.39 |
| Fetal PFOA | 1 | 0.0204 | 6.09 | 4.68 | 0.104 | 16.4 | 7.312 | 0.17 | 24 |

The bold font means remained statistically significance after adjusting for maternal age, BMI, past smoker, GA at exam, and fetal sex. EDC, endocrine disrupting chemicals; GAM, generalized additive model; BPD, biparietal diameter; HC, head circumference, AC; abdominal circumference; FL, femur length; EFW, estimated fetal weight; BPA, bisphenol-A; MEP, monoethyl phthalate; PFOA, perfluorooctanoic acid.

**FIGURE S1** Non-linear and linear association between EDCs in maternal urine and fetal cord blood. A. the non-linear association of each EDC between maternal urine and fetal cord blood samples using a generalized additive model. B.the linear association of each EDC between the two samples using a linear regression model.

EDC, endocrine disrupting chemicals; BPA, bisphenol-A; MEP, monoethyl phthalate; PFOA, perfluorooctanoic acid

**FIGURE S2** Non-linear associations between EDCs and fetal growth indices using a generalized additive model. A. Association between each EDC and biparietal diameter z-score. B. Association between each EDC and head circumference z-score. C. Association between each EDC and abdominal circumference z-score. D. Association between each EDC and Femur length z-score. E. Association between each EDC and EFW z-score.

EDC, endocrine disrupting chemicals; EFW, estimated fetal weight
