# Supplementary figures and images for: The associations between maternal and fetal exposure to endocrine-disrupting chemicals and asymmetric fetal growth restriction: a prospective cohort study
Source: Front Public Health. 2024 Apr 11;12:1351786. doi: 10.3389/fpubh.2024.1351786 (PMC11043493; doi:10.3389/fpubh.2024.1351786)

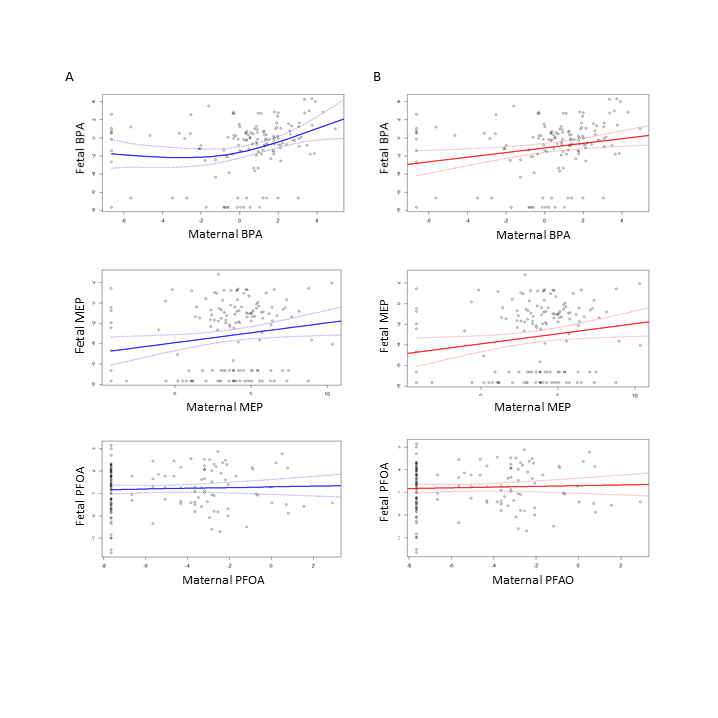

Supplement: Supplementary file 3 [file Image_1.TIF]

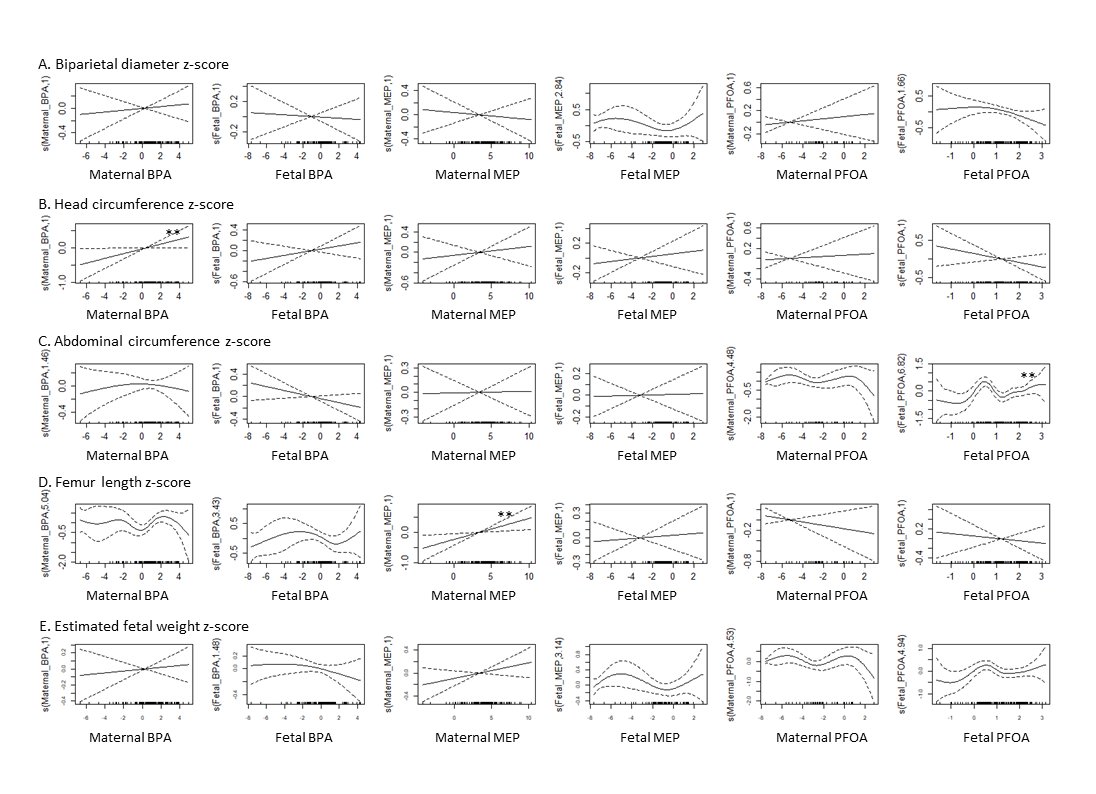

Supplement: Supplementary file 4 [file Image_2.TIF]
